# Empirical Therapy in High-Risk Hematological Patients with Carbapenem-Resistant *Enterobacterales* Colonization: Real-World Treatment Decisions and Antimicrobial Stewardship Implications

**DOI:** 10.3390/antibiotics15100992

**Published:** 2026-10-07

**Authors:** Maria Helena Parrinello, Flavia Badalucco Ciotta, Raffaele Dell’Acqua, Vincenzo Spagnuolo, Enzo Scifo, Francesca Farina, Raffaella Greco, Consuelo Corti, Silvia Carletti, Paolo Scarpellini, Antonella Castagna, Fabio Ciceri, Daniela Clerici, Marco Ripa

**Affiliations:** 1 Faculty of Medicine and Surgery, Vita-Salute San Raffaele University, 20132 Milan, Italy; parrinello.maria@hsr.it (M.H.P.);; 2 Unit of Hematology and Bone Marrow Transplantation, IRCCS San Raffaele Scientific Institute, 20132 Milan, Italy; 3 Unit of Infectious Diseases, IRCCS San Raffaele Scientific Institute, 20127 Milan, Italy; 4 Laboratory of Microbiology and Virology, IRCCS San Raffaele Scientific Institute, 20132 Milan, Italy

**Keywords:** carbapenem-resistant *Enterobacterales*, antimicrobial stewardship, hematological malignancies, febrile neutropenia, intestinal colonization, empirical antimicrobial therapy, bloodstream infection, active surveillance, metallo-β-lactamase, multidrug-resistant Gram-negative bacteria

## Abstract

Background/Objectives: Carbapenem-resistant *Enterobacterales* (CRE) colonization is a risk factor for infection in hematological patients, but only a minority of colonized individuals develop invasive disease. This creates a stewardship dilemma between ensuring prompt, appropriate empirical therapy and avoiding unnecessary exposure to novel anti-CRE agents. We aimed to describe real-world empirical treatment decisions in CRE-colonized hematological patients. Methods: Retrospective single-center observational study including adult hematological patients with documented CRE colonization between January 2021–February 2025. The primary objective was to describe the use of empirical therapy active against the colonizing CRE. Secondary objectives were to evaluate concordant infections (caused by the same species and carbapenemase mechanism as the surveillance isolate) and subsequent de-escalation of CRE-active empirical therapy. Results: Thirty-two CRE-colonized patients were included. *Klebsiella pneumoniae* was the predominant colonizing species, and metallo-β-lactamase-producing isolates accounted for 90.6% of colonizing CRE. Empirical CRE-active therapy was administered to 20 patients (62.5%). Treated patients more frequently had febrile neutropenia and earlier colonization after hematological treatment and hospital admission. Concordant infection occurred in 5 patients (15.6%), all of whom had received empirical CRE-active therapy, whereas none of the untreated patients developed concordant infection. De-escalation was performed in 9 of 20 treated patients (45%). Conclusions: In this real-world cohort, empirical CRE-active therapy was preferentially administered to colonized patients with a high-risk clinical profile. However, concordant infection occurred in a minority of patients, highlighting the stewardship challenge of balancing early appropriate therapy with prudent use of novel anti-CRE agents.

## 1. Introduction

Bloodstream infections (BSIs) caused by multidrug-resistant Gram-negative bacteria (MDR-GNB) remain a major threat in patients with hematological malignancies and in recipients of hematopoietic stem-cell transplantation or cellular therapies [1,2,3,4].

Gut colonization with MDR organisms is a well-recognized risk factor for infection, but only a minority of colonized patients actually develop infections caused by the colonizing pathogen [5,6,7,8]. The risk of bacterial translocation and invasive infection in this high-risk population is amplified by several factors including prolonged neutropenia, mucosal barrier disruption, central venous catheters, repeated exposure to broad-spectrum antibiotics, and prolonged or recurrent hospitalization.

The same clinical features that justify early appropriate antimicrobial therapy may also lead to unnecessary broad-spectrum exposure, particularly when colonization does not progress to infection. This may, in turn, promote further antimicrobial resistance, increase drug-related toxicity, and predispose patients to complications such as Clostridioides difficile infection, and undermine the goals of antimicrobial stewardship (AMS) [9].

Carbapenem-resistant *Enterobacterales* (CRE) represent one of the most challenging resistance phenotypes. A frequent and increasingly complex scenario is the management of patients who are colonized with CRE but have no evidence of invasive disease at the time of fever onset. Rectal colonization identifies a reservoir for possible subsequent infection, yet the absolute risk of progression from colonization to infection varies substantially according to host characteristics, timing after hematological treatment, neutropenia, local epidemiology, species, resistance mechanism, and clinical severity at presentation [10].

This remains a major unresolved clinical dilemma. On one hand, delayed active therapy in febrile neutropenia or sepsis caused by resistant Gram-negative bacteria can increase infection-related mortality. On the other hand, empirical use of novel agents against CRE in every colonized patient may be disproportionate when only a minority develop infection caused by the colonizing pathogen. In this context, the real-world use of surveillance data to guide empirical therapy represents an important antimicrobial stewardship question. The central issue is whether CRE colonization is being used as a dynamic risk marker within a broader clinical assessment, rather than as a binary trigger for escalation to novel anti-CRE therapy.

The aim of this study was to describe real-world empirical antimicrobial treatment decisions among high-risk hematological patients with documented CRE colonization. We specifically assessed the use of CRE-active empirical therapy, the clinical and microbiological characteristics associated with initiation of such therapy, the occurrence of subsequent concordant infections, and subsequent treatment de-escalation. We finally sought to explore the stewardship implications of empirical CRE-active therapy in this highly vulnerable population.

## 2. Materials and Methods

### 2.1. Study Design and Population

We conducted a single-center retrospective observational study including adult patients admitted to the Hematology Department of the IRCCS San Raffaele Scientific Institute (Milan, Italy) colonized by CRE, between January 2021 and February 2025.

Active surveillance for intestinal colonization was performed using rectal swabs at admission and weekly thereafter during hospitalization. The study included patients who were colonized at admission as well as during follow-up. However, patients whose rectal swab was performed and tested positive within a 5-day period prior to the date of discharge were not included.

Rectal swabs were processed at the Microbiology and Virology Laboratory by inoculation onto MacConkey agar. A 10-microgram meropenem disc was placed in the densest area of the culture; colonies growing within the zone of bacterial growth inhibition (e.g., within an area corresponding to an inhibition zone with a diameter < 30 mm) were considered worthy of further study. These bacterial colonies were then tested for carbapenemase production (KPC [*Klebsiella pneumoniae* carbapenemase], OXA-48 [Oxacillinase-48], VIM [Verona integron-encoded metallo-beta-lactamase], IMP [Imipenemase], NDM [New Delhi metallo-beta-lactamase]) using the NG-test/carba-5 immunochromatographic assay (NG Biotech SAS, Guipry-Messac, France). Subsequently, identification was performed using mass spectrometry (Vitek MS BioMérieux, Marcy-l’Étoile, France), and antibiotic susceptibility testing was conducted using the Vitek 2 BioMérieux system.

The study was conducted in accordance with the Declaration of Helsinki. Written informed consent for therapeutic procedures and for use of medical records was obtained according to institutional practice. In addition, allo-SCT (allogeneic stem-cell transplantation) recipients had previously provided written informed consent for participation in the non-interventional ALMON study, approved by San Raffaele Institutional Ethical Committee on 19 October 2007, which allowed the use of medical records and immunological studies in patients undergoing allo-SCT. ALMON study did not determine or mandate antimicrobial treatment.

### 2.2. Data Collection and Definitions

The following variables were collected: age, sex Charlson comorbidity index, underlying hematological disease, most recent and ongoing hematological therapy, timing from the last hematological treatment to colonization detection, timing from hospital admission to the first positive surveillance swab, CRE species and carbapenemase mechanism, occurrence and timing of neutropenia (including grade 4 neutropenia, defined as an absolute neutrophil count (ANC) < 0.5 × 10^9^/L according to CTCAE [Common Terminology Criteria for Adverse Events] version 5.0 [11]), microbiologically documented infection, empirical therapy active against the colonizing organism, treatment de-escalation, Clostridioides difficile infection, septic shock, infection-attributable mortality and in-hospital mortality.

For the purposes of this study, “concordant infection” was defined as a microbiologically documented infection caused by the same *Enterobacterales* species and carbapenemase mechanism identified in the preceding CRE surveillance isolate. Because strain-level genomic relatedness was not assessed, concordance was not considered proof of infection caused by the same bacterial clone or strain. For patients with multiple infections caused by the same species/resistance phenotype, only the first infection after the first positive surveillance swab was considered for the primary concordant infection analysis.

Antimicrobial therapy was initiated according to institutional guidelines, and the first-line empiric therapy for uncomplicated febrile neutropenia in patients without specific risk factors for multi-drug resistant organisms was piperacillin/tazobactam. For patients colonized by CRE, empirical therapy active against the colonizing CRE was defined as initiation of an antimicrobial regimen with expected activity against the species and carbapenemase mechanism identified in the surveillance isolate, according to local microbiological data and institutional antimicrobial practice. The decision to initiate CRE-active empirical therapy was made by the treating clinical team according to the clinical presentation and overall assessment of the patient’s risk of resistant bacterial infection. Empirical therapy was initiated at the time of the clinical episode prompting microbiological investigation; concordant infection was therefore defined according to the subsequent microbiological result. De-escalation was defined as discontinuation of CRE-active therapy or transition to an antimicrobial regimen without activity against the colonizing CRE organism when subsequent clinical or microbiological assessment did not support ongoing treatment directed against the colonizing CRE.

### 2.3. Study Aims

The primary aim of the study was to describe the management of patients colonized by CRE, with a specific focus on the use of empirical therapy active against the colonizing CRE.

The secondary aim was to describe the occurrence of concordant infection after CRE colonization, and subsequent de-escalation of CRE-active empirical therapy.

### 2.4. Statistical Analysis

Continuous variables are presented as median and interquartile range (IQR), and categorical variables as absolute numbers and percentages. Characteristics of patients who did and did not receive empirical therapy active against the colonizing CRE were compared using Chi-square or Fisher’s exact test for categorical variables and the Mann–Whitney test for continuous variables. Given the retrospective design, the limited sample size, and the small number of concordant infection events, analyses were considered exploratory and primarily descriptive. Comparisons between treatment groups were considered hypothesis-generating. No multivariable model was performed because of the limited number of outcome events. A two-sided *p*-value < 0.05 was considered statistically significant.

## 3. Results

### 3.1. Patients’ Characteristics and Hematological Context

Between January 2021 and February 2025, 969 hematological patients were admitted to the Hematology Department of our Hospital. Overall, 32 CRE-colonized patients were included. The cohort was characterized by a predominance of high-risk hematological disease and recent intensive therapies (Table 1). The majority of them were affected by acute leukemia (62.5%). The most recent hematological therapy was allo-SCT in 20 patients (62.5%), followed by chemotherapy in 6 patients (18.8%), autologous stem-cell transplantation in 3 patients (9.4%) and CAR T-cell therapy in 3 patients (9.4%). The first positive surveillance swab occurred early in relation to hematological treatment, with a median interval of 11 days (range 3–18) from the last hematological therapy.

### 3.2. CRE Species and Resistance Mechanisms

*Klebsiella pneumoniae* was the most prevalent colonizing species, detected in 14 patients (43.8%), followed by *Escherichia coli* in 9 patients (28.1%), *Citrobacter freundii* in 4 patients (12.5%), *Enterobacter cloacae* in 4 patients (12.5%) and *Klebsiella oxytoca* in 1 patient (3.1%). Metallo-β-lactamase (MBL)-producing organisms accounted for 29 of 32 isolates (90.6%). VIM was the predominant resistance mechanism, identified in 22 isolates (68.8%), followed by NDM in 7 isolates (21.9%) and KPC in 2 isolates (6.3%). One *Citrobacter freundii* isolate presented both KPC and VIM mechanisms. Data are presented in Table 2.

### 3.3. Empirical Therapy

Empirical therapy active against the colonizing pathogen (Table 3) was administered to 20 of 32 patients (62.5%). Eighteen of these patients were colonized with MBL-producing organisms and two with KPC-producing organisms.

Among patients colonized with MBL-producing organisms, the predominant regimen was ceftazidime–avibactam plus aztreonam, used in 16 of these 18 patients (88.8%), followed by ceftazidime–avibactam plus tigecycline in 1 patient (5.5%) and cefiderocol in 1 patient (5.5%). For both patients colonized by KPC-producing organisms, ceftazidime/avibactam was used.

In 16 of 20 patients (80%), the initiation of CRE-active therapy represented an escalation from a previous regimen not active against the colonizing strain.

Treatment was subsequently de-escalated in 9 of the 20 patients (45%) receiving CRE-active empirical therapy, either after identification of an alternative bacterial pathogen or when subsequent clinical and microbiological evaluation did not support the diagnosis of concordant infection. Four patients who received CRE-active empirical therapy developed *C. difficile* infection, compared to none in the group who did not receive CRE-active empirical treatment.

Compared with patients who did not receive empirical therapy active against the colonizing pathogen, those who received CRE-active empirical therapy had a shorter interval between admission and the first positive rectal swab (11 [6–18] vs. 27 [25–35] days, *p* < 0.001) and a shorter time from the last hematological treatment to colonization detection (6 [−6–11] vs. 18 [17–27] days, *p* < 0.001). They were more likely to have febrile neutropenia (19/20 [95.0%] vs. 5/12 [41.7%], *p* = 0.001), were more frequently colonized by NDM-producing strains (7/20 [35.0%] vs. 0/12, *p* = 0.008), and developed neutropenia closer to the last hematological treatment (9 [2–12] vs. 18 [14–139] days, *p* = 0.036).

### 3.4. Concordant Infections and Clinical Outcomes

Among patients included in our cohort, 5/32 (15.6%) developed concordant infections (Table 4). In all five cases, CRE-active empirical therapy was initiated at the time of the clinical episode from which the concordant infection was subsequently microbiologically identified. Conversely, none of the 12 patients who did not receive empirical CRE-active therapy were subsequently found to have an infection sustained by the colonizing pathogen. All concordant infections were caused by *K. pneumoniae*: 3 NDM-producing isolates, 1 VIM-producing isolate and 1 KPC-producing isolate, with a median of 18 days (range 0–36) after the first detection of colonization. The types of infection included 2 central line-associated BSIs (CLABSI), 2 primary BSIs, and one nosocomial pneumonia. No patient developed septic shock, and no death was directly attributed to infection-related complications.

All five patients who developed a concordant infection had additional major host- or treatment-related risk factors, including ongoing or new cytotoxic therapy, delayed engraftment, recurrent infections, graft rejection, or severe graft-versus-host disease. These observations suggest that CRE colonization occurred within a broader context of profound host vulnerability.

Overall, in-hospital mortality occurred in 4 patients (12.5%); 2 had confirmed VIM-producing *K. pneumoniae* infection, but mortality was not considered directly attributable to infection. Both had received empirical therapy active against the colonizing pathogen.

## 4. Discussion

In this retrospective cohort of CRE-colonized hematological patients, we observed that empirical CRE-active therapy was not administered uniformly. Instead, treatment was concentrated among patients with a distinct clinical and microbiological profile, including more frequent febrile neutropenia and colonization detected closer to hospitalization and recent hematological treatment. Concordant infection occurred in a minority of the overall cohort, while most patients receiving CRE-active therapy did not subsequently develop documented concordant infection. These findings illustrate the central stewardship challenge of empirical treatment in CRE-colonized hematological patients: the need to preserve prompt active therapy for patients at greatest risk while limiting unnecessary exposure to novel anti-CRE agents.

In our cohort, metallo-β-lactamase (MBL) producers accounted for the large majority of colonizing isolates (90.6%), with VIM alone responsible for nearly two-thirds (68.8%), while KPC-producing strains were rare (2/32 isolates, 6.3%, one of which co-harbored VIM). This distribution differs from the KPC-dominant picture that has historically characterized carbapenem resistance among Italian and European hematology and hematopoietic stem-cell transplant populations [3,4,8] and emphasizes the importance of local epidemiology when interpreting CRE surveillance results. The predominance of MBL-producing organisms also has direct implications for empirical treatment decisions [12,13], as the therapeutic options and antimicrobial combinations considered active against MBL-producing organisms differ from those used for KPC-producing CRE.

We observed that while 62.5% of our cohort received empirical therapy active against their colonizing pathogen, only 15.6% (5/32) developed a concordant infection. Our overall colonization-to-infection progression rate sits within the range reported in recent hematology-specific colonization cohorts [2,5,12,14,15,16], and is broadly consistent with the observation that carriage of CRE carries a substantially higher subsequent infection risk than carriage of other multidrug-resistant phenotypes [6]. Notably, our results focus on a severely immunosuppressed population, and therefore arguably more vulnerable to any given colonizing organism, than the mixed medical-surgical or ICU cohorts that dominate the existing CRE colonization literature.

A central observation of our study was that empirical CRE-active therapy was not administered uniformly to all colonized patients. Patients who ultimately received CRE-active empirical therapy were characterized by a distinct clinical and microbiological profile: a shorter interval between admission and first positive swab, a shorter interval between the last hematological treatment and colonization detection, more frequent febrile neutropenia, more frequent NDM colonization, and neutropenia developing closer to the last hematological treatment. This observation is particularly relevant from an antimicrobial stewardship perspective. Rectal surveillance, combined with clinical context, contributed to a broader clinical risk assessment that warranted prompt CRE-active coverage in selected patients, differentiating them from patients who could reasonably be managed with a narrower regimen pending culture results. This supports the central premise motivating this analysis: that surveillance data, interpreted alongside timing and severity of presentation, can help move empirical therapy away from a binary “colonized = treat with novel agents” rule and toward an individualized approach, consistent with the risk-adapted framework recommended by the 10th European Conference on Infections in Leukaemia (ECIL-10) [9] and with recent hematology cohort data showing that colonization status meaningfully improves risk stratification for subsequent bloodstream infection and can shorten time to adequate therapy when acted upon [2]. This balance is especially important because timely appropriate empirical antimicrobial therapy has been associated with improved clinical outcomes in hematological patients with severe Gram-negative bloodstream infections [17], while unnecessary broad-spectrum therapy remains a key antimicrobial stewardship concern [18]. Nevertheless, although prior reports linked inappropriate empirical therapy to worse outcomes, our cohort had no inappropriate empirical regimens against CRE, and thus cannot address that association reliably.

Notably, while 20 of 32 colonized patients (62.5%) received empirical therapy active against the colonizing organism, only 5 of those 20 (25%) went on to have a concordant infection, meaning that three in four patients who received CRE-active empirical therapy did not have, in retrospect, a confirmed concordant infection. Similar proportions were shown in previous reports focusing on empirical treatment of hematological patients colonized with CRE [13,16], although scarce data are available on patients colonized by MBL-producing *Enterobacterales*. Reassuringly, none of the 12 patients who did not receive empirical CRE-active coverage went on to develop concordant infection. These patients were characterized by a longer interval from admission to colonization detection, a longer interval from their last hematological treatment, and a significantly lower incidence of febrile neutropenia, suggesting that a strategy of withholding novel-agent coverage in lower-risk colonized patients may be feasible in selected patients. Nevertheless, a substantial amount of broad-spectrum, novel-agent antibiotic exposure in this population occurred in patients who did not ultimately need it for the organism it was aimed at. In this sense, the gap between empirical treatment and subsequent concordant infection represents a measurable consequence of diagnostic uncertainty and a relevant target for antimicrobial stewardship interventions. A similar stewardship challenge has been observed in predominantly KPC-endemic settings, where empirical ceftazidime–avibactam has been used in high-risk hematological patients with fever of unknown origin and prior CRE colonization, despite the absence of subsequent microbiological confirmation in a substantial proportion of episodes [19].

Encouragingly, treatment was de-escalated in 9 of the 20 (45%) patients empirically treated with CRE-active agents once alternative pathogens were identified or cultures did not support concordant infection, suggesting that subsequent clinical and microbiological reassessment led to de-escalation in a substantial proportion of treated patients. It is plausible that a more structured early reassessment and de-escalation strategy could further reduce exposure to CRE-active agents without compromising patients who ultimately require active treatment [20,21]. Prospective protocols incorporating predefined reassessment time points and rapid microbiological data may help reduce exposure to novel anti-CRE agents while preserving prompt treatment for patients at greatest risk of resistant infection.

Notably, in our cohort, 20% of the patients who received empirical CRE-active therapy subsequently developed CDI, while no patients developed CDI in the group of patients who did not receive CRE-active therapy. Although our small sample size limited our statistical power to demonstrate a significant difference compared to the untreated group, this observation highlights the broader collateral consequences of antimicrobial exposure in a heavily treated hematological population.

Importantly, all 5 concordant infections were due to *Klebsiella pneumoniae*. Although this observation is clinically interesting, the number of events is too small to support a species-specific treatment recommendation. Moreover, the absence of genomic sequencing prevents confirmation of strain-level relatedness between the surveillance and clinical isolates. Nevertheless, previous metagenomic studies have shown gut and bloodstream microbial isolates to be closely related [22]. The exclusive occurrence of concordant infections in patients colonized with *K. pneumoniae* should therefore be considered hypothesis-generating and warrants validation in larger cohorts.

The clinical characteristics of the five patients with concordant infection also deserve attention. All had additional major host or treatment-related risk factors, including ongoing or new chemotherapy, delayed engraftment, recurrent infections, graft rejection, or severe graft-versus-host disease. This observation supports a multidimensional interpretation of CRE colonization. In particular, the clinical significance of colonization may depend not only on the resistance mechanism or bacterial species, but also on the depth and timing of immunosuppression and the patient’s evolving hematological status.

Our study has several limitations. First, its retrospective, single-center design limits the generalizability of our findings to centers with different local epidemiologies (e.g., those dominated by KPC or OXA-48). Second, the study population was restricted to patients with documented CRE colonization. Consequently, this study cannot assess the incremental clinical value of active surveillance or compare outcomes between surveillance-positive and surveillance-negative patients. Third, the retrospective design of the study does not allow reconstruction of the individual factors that directly determined treatment decisions among treating physicians. Moreover, treatment allocation was not random and was strongly influenced by the clinical context; therefore, comparisons between patients receiving and not receiving CRE-active therapy are subject to confounding by indication. Fourth, the sample size is relatively small, which precluded our ability to perform multivariable logistic regression analyses to identify independent risk factors for progression from colonization to infection. Finally, we did not perform genomic sequencing to confirm clonal identity between the colonizing rectal strains and the subsequent clinical bloodstream isolates, though species concordance and identical resistance profiles strongly suggest translocation. Despite these limitations, our study provides valuable real-world data on the integration of active microbiological surveillance and stewardship in a highly vulnerable patient population. Rather than triggering reflexive, broad-spectrum escalation, CRE colonization should be used as a dynamic biomarker to be interpreted alongside the patient’s hematological status, clinical stability, and the specific colonizing species.

## 5. Conclusions

In this single-center retrospective study, empirical therapy active against the colonizing CRE was preferentially used in patients with a clinically distinct high-risk profile, suggesting that surveillance results were incorporated into individualized treatment decisions in routine practice. However, most CRE-active empirical treatment courses were not followed by documented concordant infection, highlighting the antimicrobial stewardship burden of empirical uncertainty in this population. These findings support the use of CRE surveillance as a component of clinical risk assessment for empirical therapy, rather than as an automatic indiscriminate trigger for novel anti-CRE treatment in every colonized patient. Prompt reassessment of clinical and microbiological data, together with timely de-escalation or discontinuation when appropriate, remains essential to balance the need for early adequate therapy with the preservation of novel anti-CRE agents.

We argue that multidisciplinary AMS must be central in guiding antibiotic decisions in colonized hematological patients. Novel anti-CRE agents represent a critical therapeutic advance against MDR-GNB, but their empirical use in colonized patients should be cautious, carefully individualized, and framed within the principles of AMS. Future research should focus on strategies that help clinicians dynamically tailor empirical therapy according to patient characteristics, evolving local epidemiology, and ongoing microbiological evidence.

## Figures and Tables

**Table 1:** Patients’ characteristics.

Characteristics	Overall (*n* = 32)
Age, years	57 (50–64)
Sex, female	8 (25.0%)
Charlson comorbidity index	3 (2–5)
Hematological disease	
Acute leukemia	20 (62.5%)
Lymphoma	8 (25.0%)
MPN or MDS	3 (9.4%)
Other	1 (3.1%)
Ongoing line of hematological therapy	
1	16 (50.0%)
2	10 (31.3%)
≥3	6 (18.8%)
Last hematological therapy	
CT	6 (18.8%)
Allo-SCT	20 (62.5%)
Auto-SCT	3 (9.4%)
CAR-T	3 (9.4%)
Conditioning regimen for Allo-SCT (*n* = 20)	
MAC	11 (55.0%)
RIC	9 (45.0%)
PTCY-based GVHD prophylaxis (*n* = 20)	18 (90.0%)
Allogeneic donor for allo-SCT (*n* = 20)	
MRD	4 (20.0%)
MUD	10 (50.0%)
HAPLO	4 (20.0%)
CB	2 (10.0%)
Allo-SCT source (*n* = 20)	
PB	17 (85.0%)
BM	1 (5.0%)
CB	2 (10.0%)
Type of CT (*N* = 6)	
Induction	2 (33.3%)
Salvage/Reinduction	4 (66.7%)
Reason for admission	
Treatment	29 (90.6%)
Complications	3 (9.4%)

Data are presented as median (IQR) or *n* (%), as appropriate. Abbreviations: Allo-SCT, allogeneic stem-cell transplantation; auto-SCT, autologous stem-cell transplantation; BM, bone marrow; CAR-T, chimeric antigen receptor T-cell therapy; CB, cord blood; CT, chemotherapy; GVHD: graft-versus-host disease; HAPLO, haploidentical donor; MAC, myeloablative conditioning; MDS, myelodysplastic syndrome; MPN, myeloproliferative neoplasm; MRD, matched related donor; MUD, matched unrelated donor; PB, peripheral blood; PTCY, post-transplant cyclophosphamide; RIC, reduced-intensity conditioning.

**Table 2:** Microbial isolates.

Microbial Isolate		*N* (%)
*Klebsiella pneumoniae*		14 (43.8%)
	KPC	1 (7.1%)
	VIM	7 (50.0%)
	NDM	6 (42.9%)
*Escherichia coli*		9 (28.1%)
	KPC	1 (11.1%)
	VIM	7 (77.8%)
	NDM	1 (11.1%)
*Citrobacter freundii*		4 (12.5%)
	VIM	3 (75.0%)
	KPC + VIM	1 (25.0%)
*Enterobacter cloacae*		4 (12.5%)
	VIM	4 (100%)
*Klebsiella oxytoca*		1 (3.1%)
	VIM	1 (100%)

Data are presented as *n* (%). Abbreviations: KPC, *Klebsiella pneumoniae* carbapenemase; NDM, New Delhi metallo-β-lactamase; VIM, Verona integron-encoded metallo-β-lactamase.

**Table 3:** Patients’ characteristics according to empirical therapy active against colonizing pathogen.

Characteristics	Overall (*n* = 32)	No Empirical Therapy Active Against Colonizing Pathogen	Empirical Therapy Active Against Colonizing Pathogen	*p*-Value
		(*n* = 12)	(*N* = 20)	
Age, years	57 (50–64)	55 (41–68)	58 (51–63)	0.893
Sex, female	8 (25.0%)	2 (16.7%)	6 (30.0%)	0.676
Charlson comorbidity index	3 (2–5)	3 (1–5)	3 (3–5)	0.272
Hematological disease				0.342
Acute leukemia	20 (62.5%)	7 (58.3%)	13 (65.0%)	
Lymphoma	8 (25.0%)	2 (16.7%)	6 (30.0%)	
MPN or MDS	3 (9.4%)	2 (16.7%)	1 (5.0%)	
Other	1 (3.1%)	1 (8.3%)	0 (0.0%)	
Ongoing line of hematological therapy				0.707
1	16 (50.0%)	5 (41.7%)	11 (55.0%)	
2	10 (31.3%)	4 (33.3%)	6 (30.0%)	
≥3	6 (18.8%)	3 (25.0%)	3 (15.0%)	
Last hematological therapy				0.050
CT	6 (18.8%)	(0.00%)	6 (30.0%)	
Allo-SCT	20 (62.5%)	10 (83.3%)	10 (50.0%)	
Auto-SCT	3 (9.4%)	0 (0.0%)	3 (15.0%)	
CAR-T	3 (9.4%)	2 (16.7%)	1 (5.0%)	
Conditioning regimen for Allo-SCT (*n* = 20)				0.999
MAC	11 (55.0%)	5 (50.0%)	6 (60.0%)	
RIC	9 (45.0%)	5 (50.0%)	4 (40.0%)	
PTCY-based GVHD prophylaxis (*n* = 20)	18 (90.0%)	10 (100.0%)	8 (80.0%)	0.474
Allogeneic donor for allo-SCT (*n* = 20)				0.221
MRD	4 (20.0%)	3 (30.0%)	1 (10.0%)	
MUD	10 (50.0%)	6 (60.0%)	4 (40.0%)	
HAPLO	4 (20.0%)	1 (10.0%)	3 (30.0%)	
CB	2 (10.0%)	0 (0.0%)	2 (20.0%)	
Allo-SCT source (*n* = 20)				0.217
PB	17 (85.0%)	9 (90.0%)	8 (80.0%)	
BM	1 (5.0%)	1 (10.0%)	0 (0.0%)	
CB	2 (10.0%)	0 (0.0%)	2 (20.0%)	
Type of CT (*N* = 6)				NA
Induction	2 (33.3%)	0 (0.0%)	2 (33.3%)	
Salvage/Reinduction	4 (66.7%)	0 (0.0%)	4 (66.7%)	
Reason for admission				0.999
Treatment	29 (90.6%)	11 (91.7%)	18 (85.0%)	
Complications	3 (9.4%)	1 (8.3%)	2 (10.0%)	
Microbial isolate				**0.040**
*Escherichia coli*	9 (28.1%)	2 (16.7%)	7 (35.0%)	
*Klebsiella* spp.	15 (46.9%)	4 (33.3%)	11 (55.0%)	
Other	8 (25.0%)	6 (50.0%)	2 (10.0%)	
Mechanism of resistance				**0.036**
KPC	2 (6.3%)	0 (0.0%)	2 (10.0%)	
KPC + VIM	1 (3.1%)	1 (8.3%)	(0.00%)	
NDM	7 (21.9%)	(0.00%)	7 (35.0%)	
VIM	22 (68.8%)	11 (91.7%)	11 (55.0%)	
Febrile neutropenia	24 (75.0%)	5 (41.7%)	19 (95.0%)	**0.001**
G4 (*N* = 24)	23 (94.7%)	5 (100.0%)	18 (94.7%)	0.600
Septic shock	2 (6.3%)	0 (0.0%)	2 (10.0%)	0.516
Microbiologically documented infection by any pathogen	24 (75.0%)	5 (41.7%)	19 (95.0%)	**0.001**
Microbiologically documented infection due to colonizing pathogen	5 (15.6%)	0 (0.0%)	5 (25.0%)	0.130
*C. difficile* infection	4 (12.5%)	0 (0.0%)	4 (20.0%)	0.271
In-hospital mortality	4 (12.5%)	0 (0.0%)	4 (20.0%)	0.274
Days from last hematological therapy to swab	11 (3–18)	18 (17–27)	6 (−6–11)	**<0.001**
Days from admission to swab	18 (7–27)	27 (25–35)	11 (6–18)	**<0.001**
Days from last hematological therapy to neutropenia (*n* = 24)	10 (4–20)	18 (14–139)	9 (2–12)	**0.036**
Days from swab to neutropenia (*n* = 24)	2 (0–10)	0 (0–19)	0 (0–10)	0.945
Days from swab to any first microbiologically documented infection (*n* = 24)	8 (6–19)	8 (7–24)	8 (6–17)	0.446
Days from swab to infection due to colonizing pathogen (*n* = 5)			24 (3–42)	
Days from admission to discharge (*n* = 28)	47 (37–64)	44 (38–62)	51 (36–70)	0.909
Days from swab to discharge (*n* = 28)	25 (16–42)	15 (12–23)	37 (25–46)	**0.001**
Days from swab to death (*n* = 4)			54 (21–63)	

Continuous variables are presented as median (IQR) and categorical variables as *n* (%). Comparisons were performed using the Mann–Whitney U test for continuous variables and Pearson’s χ^2^ test or Fisher’s exact test, as appropriate. All *p*-values are two-sided; *p* < 0.05 was considered statistically significant, and are shown in bold. Given the exploratory nature of the study, *p*-values should be interpreted descriptively. Abbreviations: Allo-SCT, allogeneic stem-cell transplantation; auto-SCT, autologous stem-cell transplantation; BM, bone marrow; CAR-T, chimeric antigen receptor T-cell therapy; CB, cord blood; CT, chemotherapy; HAPLO, haploidentical donor; GVHD: graft-versus-host disease; KPC, *Klebsiella pneumoniae* carbapenemase; MAC, myeloablative conditioning; MDS, myelodysplastic syndrome; MPN, myeloproliferative neoplasm; MRD, matched related donor; MUD, matched unrelated donor; NDM, New Delhi metallo-β-lactamase; PB, peripheral blood; PTCY, post-transplant cyclophosphamide; RIC, reduced-intensity conditioning; VIM, Verona integron-encoded metallo-β-lactamase.

**Table 4:** Patients with concordant infection.

Patient	Diagnosis	Colonizing Pathogen	Resistance Mechanism	Last Hematological Therapy	Days from Last Hematological Therapy to Swab Positivity	Days from Swab Positivity to Infection	Type of Infection	Grade IV Neutropenia at Infection Onset	Other Risk Factors for Infection	Antibacterial Therapy	Resolution of Infection
01	Lymphoma	*K. pneumoniae*	NDM	Chemotherapy	157	36	CLABSI	Yes	Concomitant new chemotherapy due to disease relapse	C/A + aztreonam	Yes
02	Acute leukemia	*K. pneumoniae*	NDM	Chemotherapy	6	0	HAP	Yes	Delayed engraftment, recurrent infections	C/A + aztreonam	Yes
03	Lymphoma	*K. pneumoniae*	NDM	Chemotherapy	3	6	Primary BSI	Yes	Relapse	C/A + aztreonam	Yes
04	Acute leukemia	*K. pneumoniae*	VIM	AlloSCT	10	23	Primary BSI	Yes	Graft rejection	C/A + aztreonam	Yes
05	Acute leukemia	*K. pneumoniae*	KPC	AlloSCT	45	24	CLABSI	Yes	Immunosuppression related to refractory acute graft-versus-host disease	C/A	Yes

Concordant infection was defined as infection caused by the same *Enterobacterales* species and carbapenemase mechanism identified in the preceding surveillance isolate. Genomic sequencing was not performed; therefore, concordance does not imply strain-level identity. Abbreviations: AlloSCT, allogeneic stem-cell transplantation; BSI, bloodstream infection; C/A, ceftazidime–avibactam; CLABSI, catheter-associated bloodstream infection; HAP, hospital-acquired pneumonia; KPC, *Klebsiella pneumoniae* carbapenemase; NDM, New Delhi metallo-β-lactamase; VIM, Verona integron-encoded metallo-β-lactamase.

## Data Availability

The original contributions presented in this study are included in the article. Further inquiries can be directed to the corresponding authors.

## Abbreviations

The following abbreviations are used in this manuscript:

AlloSCT	Allogeneic stem-cell transplantation;
AutoSCT	Autologous stem-cell transplantation
AMS	Antimicrobial stewardship
ANC	Absolute neutrophil count
BM	Bone marrow
BSI	Bloodstream infection
CAR-T	Chimeric antigen receptor T-cell therapy
C/A	Ceftazidime–avibactam
CB	Cord blood
CDI	Clostridioides difficile infection
CLABSI	Central line-associated bloodstream infection
CRE	Carbapenem-resistant *Enterobacterales*
CT	Chemotherapy
CTCAE	Common Terminology Criteria for Adverse Events
ECIL	European Conference on Infections in Leukaemia
GVHD	Graft-versus-host disease
HAP	Hospital-acquired pneumonia
HAPLO	Haploidentical donor
HSCT	Hematopoietic stem-cell transplantation
IQR	Interquartile range
KPC	*Klebsiella pneumoniae* carbapenemase
MAC	Myeloablative conditioning
MBL	Metallo-β-lactamase
MDS	Myelodysplastic syndrome
MDR	Multidrug-resistant
MDR-GNB	Multidrug-resistant Gram-negative bacteria
MPN	Myeloproliferative syndrome
MRD	Matched related donor
MUD	Matched unrelated donor
NDM	New Delhi metallo-β-lactamase
PB	Peripheral blood
PTCY	Post-transplant cyclophosphamide
RIC	Reduced-intensity conditioning
SCT	Stem-cell transplantation
VIM	Verona integron-encoded metallo-β-lactamase
